# Reduced bone formation markers, and altered trabecular and cortical bone mineral densities of non-paretic femurs observed in rats with ischemic stroke: A randomized controlled pilot study

**DOI:** 10.1371/journal.pone.0172889

**Published:** 2017-03-09

**Authors:** Karen N. Borschmann, Sarah S. Rewell, Sandra Iuliano, Ali Ghasem-Zadeh, Rachel A. Davey, Heidi Ho, Peta N. Skeers, Julie Bernhardt, David W. Howells

**Affiliations:** 1 School of Allied Health, La Trobe University, Bundoora, Australia; 2 Stroke Division, The Florey Institute of Neuroscience and Mental Health, Heidelberg, Australia; 3 NHMRC Centre for Research Excellence in Stroke Rehabilitation and Recovery, Melbourne, Australia; 4 Department of Endocrinology, Austin Health, University of Melbourne, Heidelberg, Australia; 5 Department of Medicine, Austin Health, University of Melbourne, Heidelberg, Australia; 6 University of Tasmania, School of Medicine, Faculty of Health, Hobart, Australia; Indiana University Purdue University at Indianapolis, UNITED STATES

## Abstract

**Background:**

Immobility and neural damage likely contribute to accelerated bone loss after stroke, and subsequent heightened fracture risk in humans.

**Objective:**

To investigate the skeletal effect of middle cerebral artery occlusion (MCAo) stroke in rats and examine its utility as a model of human post-stroke bone loss.

**Methods:**

Twenty 15-week old spontaneously hypertensive male rats were randomized to MCAo or sham surgery controls. Primary outcome: group differences in trabecular bone volume fraction (BV/TV) measured by Micro-CT (10.5 micron istropic voxel size) at the ultra-distal femur of stroke affected left legs at day 28. Neurological impairments (stroke behavior and foot-faults) and physical activity (cage monitoring) were assessed at baseline, and days 1 and 27. Serum bone turnover markers (formation: N-terminal propeptide of type 1 procollagen, PINP; resorption: C-terminal telopeptide of type 1 collagen, CTX) were assessed at baseline, and days 7 and 27.

**Results:**

No effect of stroke was observed on BV/TV or physical activity, but PINP decreased by -24.5% (IQR -34.1, -10.5, *p* = 0.046) at day 27. In controls, cortical bone volume (5.2%, IQR 3.2, 6.9) and total volume (6.4%, IQR 1.2, 7.6) were higher in right legs compared to left legs, but these side-to-side differences were not evident in stroke animals.

**Conclusion:**

MCAo may negatively affect bone formation. Further investigation of limb use and physical activity patterns after MCAo is required to determine the utility of this current model as a representation of human post-stroke bone loss.

## Introduction

Within 12 months of stroke, human adult fracture risk is increased up to 7-fold that of age-matched controls[1], and recovery from fracture is poorer in those with history of stroke[2]. Despite these serious sequelae of stroke, there are limited evidence-based recommendations to reduce post-stroke fracture risk[3]. Increased bone resorption within days of bed rest, and accelerated loss of bone mineral density (BMD) within months of stroke—particularly in stroke affected (paretic) limbs–suggests that the early sub-acute post-stroke period (<3 months) is likely the most opportune therapeutic window[4]. The detailed assessments of skeletal micro-structural and cellular changes are not always possible in humans within this time period, nor are pre-stroke measures possible. In contrast, in non-stroke animal models, measurable site-specific bone loss [5–7] occurs within days of hind-limb immobilization.

In humans, immobility[8], motor impairment[9], muscle weakness and disuse of paretic limbs[10] are associated with post-stroke bone loss. Furthermore, evidence from non-stroke animal models[11–13] demonstrate brain and central nervous system regulation of bone turnover, suggesting that stroke induced neural damage may directly increase bone loss regardless of level of physical activity[12]. To our knowledge, the skeletal effect of stroke has not been examined in animals. In a commonly employed stroke model in rats—middle cerebral artery occlusion (MCAo)—sensorimotor impairments have been observed for more than four weeks [14], which suggests that it may be a suitable model for human bone loss post-stroke. However, in contrast with humans who are often sedentary after stroke [15], physical activity has been reported to stay the same[16] or increase [17,18] in rats after stroke. The aim of this pilot study was to examine the utility of MCAo in rats as a model of the skeletal effects of stroke in humans. To align common co-morbidities between animals in this study and human stroke survivors, spontaneously hypertensive rats were examined. The primary hypothesis was that stroke would lead to a reduction in trabecular bone volume fraction (BV/TV) at the ultra-distal femur site in the paretic hindlimb 28 days after MCAo.

## Methods

This was a pilot randomized, controlled study with blinded outcome assessment. Twenty spontaneously hypertensive male rats (strain SHR/NCrlArc) aged 11 weeks sourced from the Animal Resource Centre (Canning Vale, Australia) were acclimatized in our facility for one month (Austin Health, Heidelberg, Australia). Cages contained sawdust, cardboard boxes and nesting paper, with food and water available at all times. Light-dark cycle was 12-hour (light from 7am to 7pm). Prior to surgery, animals were separated into individual cages for the study duration. At surgery, animals were aged 15 weeks and mean body weight was 320.8g (SD 15.3). All procedures in this study were approved by animal research ethics committees of La Trobe University (Bundoora, Australia) and Austin Health (Heidelberg, Australia) and performed in accordance with institutional and national guidelines (Australian code of practice for the care and use of animals for scientific purposes, 7th edition, 2004). The study is reported in accordance with the ARRIVE guidelines [19].

Animals were randomized (12:8) to stroke or sham surgery via random numbers generated on an Excel spreadsheet by an assistant not involved in surgical procedures. The MCA thread occlusion model using the methods of Longa[18]with modifications by Spratt and colleagues [20]was used to induce stroke resulting in paralysis of animals’ left side. Under anesthetic (Isoflurane inhalation by nose cone, dose/ volume: 5% induction; 2% maintenance delivered in oxygen/air mix), a silicone coated suture of 0.35mm diameter was inserted approximately 18mm through a stump created from the external carotid artery to occlude the right MCA for 90 minutes before withdrawal of the thread to allow reperfusion. Control animals underwent identical dissection of the MCA, up to the point that vascular clips were applied but no occlusion was induced. Animal allocation (stroke or sham) was revealed to the surgeon immediately prior to the application of vascular clips.

Ethically approved standard operating procedures related to humane endpoints were followed: Animals that are moribund, unresponsive or unconscious once anaesthesia has worn off, or have blue extremities indicating poor heart or lung function should be euthanized immediately. Other pre-specified reasons for euthanasia are: status epilepticus for >24 hours, wound discharge indicating infection for >2 days or failure to reverse weight loss within two weeks of nursing for hydration and nutrition.

Animals were closely monitored post-operatively for neurological behaviour, general appearance, wound, eating and drinking, vocalisation, faeces and weight loss. Animals were monitored multiple times per day for the first week, then daily thereafter. Post operatively animals were maintained in a cage that contained a warm zone. All animals received 3ml warmed saline sub-cutaneously directly after surgery and on day 1 to alleviate potential dehydration. Animals are also supplemented with a soft food mixture of baby rice, protein supplement and flavoured topping to encourage eating over the first week post-surgery.

All animals were given analgesia (buprenorphine 20μg/kg) during and for three days after surgery. On day 28, animals were overdosed with isoflurane inhalation prior to tissue collection. Femurs had soft tissue excised, were fixed in 10% formalin then stored at 4° Celsius in 70% ethanol prior to analysis.

### Outcomes

#### Trabecular and cortical bone compartments: Volumetric densities and microstructure

Micro-computed tomography (Micro-CT, Viva CT40; Scanco Medical, Bassersdorf, Switzerland) was used to determine trabecular and cortical bone micro-structure. This technique uses a low radiation dose (X-ray beam energy of 55kVp and intensity of 114μA, integration time of 100ms) to produce high-resolution images (10.5 μm). Image processing was undertaken as previously described (Chiang et al., 2009). Trabecular variables were assessed at the metaphysis of the ultra-distal femur. At this site, 630 transverse image slices were acquired then images were visually inspected to locate the first slice that was void of primary spongiosa. 110 slices proximal to that slice were analyzed, giving a total length of region of interest of 1.115 mm. Based on previous research undertaken by investigator AGZ [21], the same imaging threshold was applied for all animals to delineate bone from soft tissue.

The primary outcome was trabecular bone volume fraction (BV/TV) of left femurs (note that it was the left side of animals that was stroke affected). Secondary outcomes were: trabecular thickness (Tb.Th, mm), trabecular number (Tb.N, 1/mm), trabecular separation (Tb.Sp, mm), volumetric bone mineral density (vBMD, mgHA/cm^3^), tissue mineral density(TMD, mgHA/cm^3^), bone volume (BV, mm^3^), total volume (TV, mm^3^), connectivity density (Conn.D, 1/mm^3^) and structural model index (SMI, an indicator of the structure of trabeculae (parallel plates vs cylindrical rods) based on the surface convexity of trabecular bone) [22]. Semi-automated segmentation of trabecular and cortical bone at the distal femur site was undertaken using the manufacture’s software (version V6.5–3) [7].

Cortical bone variables were measured at the femur mid-shaft and ultra-distal end. The mid-shaft was located by viewing μ-CT scout images. Fifty-five transverse image slices were made either side of the mid-shaft site (i.e. 110 slices in total). As the mid-shaft is predominantly cortical bone and marrow cavity, cortical bone edges were detected by the manufacturer’s software, with minimal manual segmentation required. The following cortical bone variables were derived: bone volume (BV, mm^3^), total volume (TV, mm^3^), cortical thickness (Ct. Th, mm), cortical area (Ct.Ar, mm^2^), volumetric bone mineral density (vBMD, mgHA/cm^3^) and tissue mineral density (TMD, mgHA/cm^3^).

#### Bone turnover markers

Tail vein blood samples were collected at baseline and days 7 and 27 after overnight fast and were assayed by ELISA for markers of bone resorption (Serum C-terminal telopeptide of type 1 collagen, CTX, RatLaps, Immunodiagnostic Systems, IDS Ltd, CV 5.6% to 9.2%) and bone formation (N-terminal propeptide of type 1 procollagen, P1NP, IDS Ltd, intra assay CV 5.0% to 7.4%).

#### Brain ischemia

Brains were collected at day 28 and visually inspected for infarct prior to fixation in 10% formalin. Specimens were then sliced into 2mm coronal section blocks and inspected for intra-cortical infarcts.

#### Neurological impairments and activity

Body weight (grams) was recorded throughout the study, and the following tests were undertaken at baseline, and days 1–2 and 27. *Behavior tests*: A battery of tests [23] was used to assess neurological behavior on a 0–5 scale: greater impairment indicated by higher scores. Impairments were noted if the left forelimb flexed or the torso rotated consistently when the animal was lifted off the bench by the tail, lost balance when pushed laterally, and if the animal was observed to be less mobile than usual.*Foot-faults*: This is a sensorimotor test of limb placement during walking[24]. Animals were video- recorded [HD Portable DVR, Proximus Products, Miami, FL, USA] walking across an enclosed horizontal 100cm ladder with 10cm long rungs spaced 4.5 cm apart. The number of times the left hindlimb slipped off or missed a rung was recorded as a “foot-fault”. Animals were recorded three times and results are expressed as average percentage of foot-faults per total number of steps taken[24].

This method is commonly used for testing hind limb neurological impairments, but reliability of the method has not been reported and there are inconsistencies in scoring of foot-faults [25,26]. Therefore, inter-tester reliability was undertaken for the method used in the current study. A standardized description of how to code foot-faults was developed based on the description by Metz & Whishaw (2009) [24]^25^. Two blinded assessors independently scored videos of two animals. Scores were discussed to reach agreement. Both assessors then independently scored all videos; analysis of inter-tester reliability is described below. *Spontaneous physical activity*: An animal behavior ethogram was completed to record physical activity. Animals were video-recorded in their home cages for one hour in the afternoon [HD Portable DVR, Proximus Products, Miami, FL, USA and Hard disk camcorder gz-mg20AA, JVC, Victor Company, Yokohama, Japan]. The scan sampling technique was used to document the activity that animals were undertaking every five minutes [27]. Behaviors were coded as “active” (climbing, grooming, eating, interacting with objects, seeking neighboring animals) or “not active” (sleeping and resting). To indicate anti-gravity motor activity, it was noted whether animals stood on their hind-limbs with their forepaws lifted off the ground during recordings.

### Sample size calculation

This study was the first to examine the skeletal effect of MCAo; therefore data were not available to calculate a sample size estimate for the primary outcome (i.e. changes in trabecular bone volume fraction BV/TV). Based on previous work using MCAo [20], a sample size calculation (power = 0.8; alpha = 0.05) estimated that 10 animals per group is required to detect a difference of 40% in brain tissue loss resulting from ischemic stroke assessed at day 28 post-stroke (32.76 ± 7.82%). Randomization occurred in a ratio of 3:2 stroke: sham allowing for expected stroke-related deaths.

### Statistical analysis

Available data from all rats were included in the intention-to-treat primary analyses. A sub-analysis was also undertaken including only animals with observed infarct that survived to day 28. Due to small sample sizes and data distribution, non-parametric analyses were undertaken. The percentage difference of bone variables between left and right legs were calculated: [(right side–left side) /left side) x 100]. Wilcoxon rank-sum test was used to compare raw values for each limb and side-to-side differences between stroke and control animals.

Between group comparisons (stroke vs control) and within group changes from baseline values of secondary outcomes (neurological behavior, foot-fault test, spontaneous physical activity, and percent change in bone turnover markers) were made by Wilcoxon rank-sum test. Associations between bone variables and secondary outcomes were tested with Spearman’s Rho. Because this was an exploratory study, the significance level of *p* = 0.05 was not adjusted for multiple comparisons. Inter-tester reliability of the foot-fault test was assessed by correlating the scores from two independent assessors using Spearman’s Rho. Analyses were performed using STATA statistical software (StataCorp LP, Texas, USA).

## Results

### Survival, stroke characteristics and body weight

**Fig 1:**
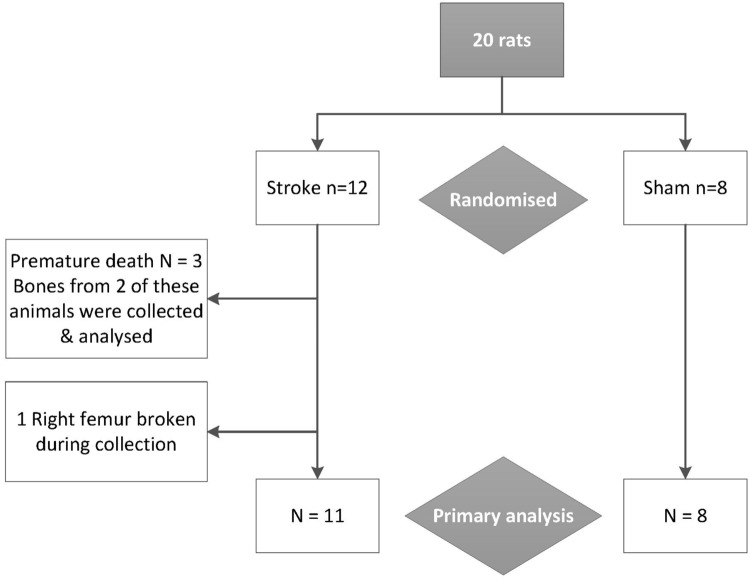
Retention of animals and bones through study. Bones from the animal that died during surgery were not collected. Bones from the other two animals that died prematurely are included in the primary analysis.

Seventeen rats survived to day 28 (Fig 1); all that died were randomized to stroke. One animal died intra-operatively whilst anaesthetised, one animal was euthanized due to an uncontrolled bleed at the incision site and one experienced an uncontrollable bleed whilst anaesthetised for examination of its incision site. Tissues were not collected for the animal that died in surgery, tissues for the other two premature deaths were collected and analyzed. The right femur of another stroke rat was broken during collection so was unavailable for scanning.

Ischemic damage to brain tissues was observed in 10/11 stroke animals that had tissues collected; no infarcts were observed in controls. All animals were neurologically intact at baseline, and control animals did not display impairments on the behavior test battery throughout the study. Stroke animals displayed impairments at 24 hours (median = 2.5, IQR [0, 3.5], *p* = 0.01) and day 27 (0, IQR [0, 0.5], *p* = 0.047).

At baseline, mean body weight was 320.8 ± 15.3g, with no difference between groups. Both groups lost weight in the first 24 hours (stroke = -21% ± 6.5 *p <* 0.001, control = -18% ± 7.9, *p <* 0.001), but surviving animals regained lost weight by day 28. Weight loss did not differ between groups throughout the study.

### Foot-faults

There was no difference in the percentage of foot-faults between stroke and control animals at baseline, acute (day 1–2) or late (day 27–28) time points, (data not shown). Baseline data were missing for the first five animals (two stroke and three controls) due to technical issues. The inter-rater concordance correlation coefficient of foot-fault counts was *r* = 0.66 (*p<* 0.001), indicating good agreement between raters [28]. The slope of the reduced major axis was 0.85, indicating that the lines diverged as the percentage of foot-faults per step increased, suggesting a proportional bias.

### Physical activity: Cage monitoring

There was no significant difference between stroke and control animals in the proportion of observations of active at baseline, acute or late assessments. At no time point were changes observed from baseline in either group in cage activity or the proportion of animals that stood up on two legs.

### Bone mineral density and structure

**Fig 2:**
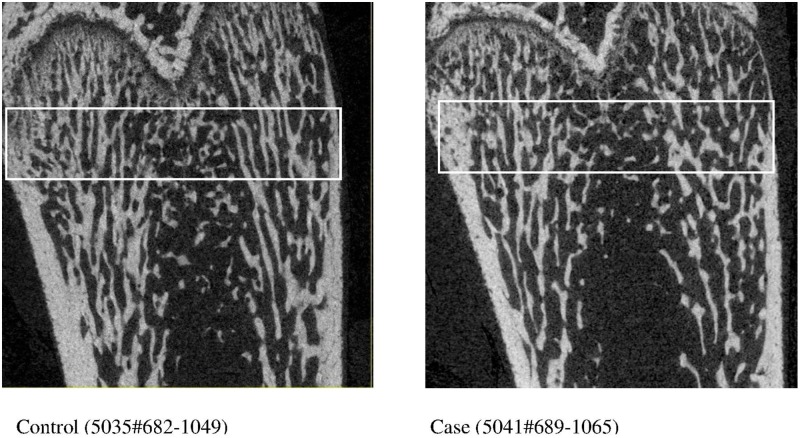
Coronal section micro-CT images of ultra-distal femurs of left (non-paretic) legs of animals with sham surgery control and middle cerebral occlusion stroke (case). White boxes indicate the regions of analysis for bone density and bone micro-structural parameters. Image on left = Control (5035#682–1049). Image on right = Case (5041#689–1065).

No significant difference in trabecular BV/TV at the ultra-distal femur of the left legs was observed between stroke and control animals using both intention-to-treat analysis (i.e. all animals), or when limiting the analysis to stroke animals with observed infarct that survived to day 28 and control animals (stroke n = 8, control n = 8), Table 1. There were group differences observed in cortical bone parameters at the ultra-distal femur site, but not at the mid-shaft, Table 2. Significant group differences were observed in between-limb comparisons of cortical TV, BV and TMD (p = 0.03, 0.02 and 0.02 respectively). These between-limb changes can be explained by increased cortical TV in left femurs (p = 0.05, stroke affected side) and reductions in BV (trend, p = 0.06) and vBMD (p = 0.02) in the right femur (non-affected limb) of animals with stroke. Inversely with changes in cortical TV, trabecular TV was higher in left legs in controls, but this difference was reduced in animals with stroke: a significant between-group comparison (p = 0.02), Table 1. Coronal section micro-CT images of ultra-distal femurs of left legs of animals with sham surgery control and MCAo are shown in Fig 2.

**Table 1:** Trabecular bone variables: Ultra-distal femurs of stroke and control rats.

	Stroke			Control			p-value#		
	Left / Paretic	Right / Non-paretic	STS % Diff^	Left	Right	STS % Diff^	Left	Right	STS
	N = 11			N = 10		N = 10		N = 8	
BV/TV	26.3 (21.9, 26.7)	25.5 (23.4, 27.4)	0.6 (-9.1, 8.2)	26.2 (25.0, 28.1)	25.3 (23.2, 28.1)	-2.4 (-7.8, 0.4)	0.48	0.93	0.42
TV	13.8 (13.1, 14.3)	13.9 (13.4, 14.4)	3.5 (-1.7, 9.1)	13.3 (13.0, 14.0)	12.8 (12.6, 13.3)	-2.8 (-5.6, -1.0)	0.48	0.01	0.02
BV	3.7 (2.8, 3.8)	3.6 (3.3, 3.9)	2.9 (-9.8, 16.5)	3.6 (3.3, 3.8)	3.4 (3.0, 3.7)	-7.6 (-12.9, -2.4)	0.93	0.48	0.16
vBMD	304.9 (257.5, 313.6)	295.7 (273.8, 315.5)	-1.6 (-8.7, 6.4)	304.2 (290.5, 325.7)	297.2 (275.2, 329.6)	-2.2 (-6.7, 0.4)	0.53	0.72	0.66
TMD	905.8 (897.4, 916.5)	892.3 (888.7, 895.5)	-1.5 (-3.0, 0.4)	909.6 (903.3, 917.0)	907.5 (900.9, 911.6)	-0.7 (-1.3, 0.2)	0.80	0.03	0.13
Conn. D	211.4 (177.8, 227.1)	194.1 (182.9, 230.0)	1.4 (-8.5, 7.6)	210.6 (188.4, 234.4)	206.0 (195.9, 226.2)	-5.2 (-9.6, 8.8)	0.59	0.86	0.59
SMI	1.33 (1.26, 1.55)	1.33 (1.19, 1.42)	-7.1 (-11.3, 9.0)	1.3 (1.2, 1.4)	1.4 (1.3, 1.6)	4.4 (0.6, 13.7)	0.86	0.37	0.25
Tb. N	4.8 (4.1, 5.1)	4.8 (4.6, 5.0)	1.5 (-1.7, 11.1)	4.9 (4.7, 5.2)	4.8 (4.6, 5.1)	-0.9 (-7.5, 3.6)	0.25	1.0	0.29
Tb. Th	67.6 (66.8, 6.99)	66.7 (64.9, 68.2)	-2.2 (-4.9, 2.1)	68.9 (68.2, 69.9)	68.7 (66.2, 72.3)	-0.9 (-2.1, 2.1)	0.42	0.25	0.53
Tb. Sp	0.19 (0.18, 0.24)	0.19 (0.18, 0.20)	-0.9 (-15.0, 1.3)	0.187 (0.176, 0.199)	0.192 (0.179, 0.205)	1.8 (-4.7, 8.1)	0.29	1.0	0.25

*Note*. Median values (IQR) presented.

^ Side-to-side % difference; calculated [(right leg–left leg)/ left leg] x 100

^#^Between-group comparison: Wilcoxon rank-sum test

vBMD = volumetric bone mineral density, mg HA/cm^3^; BV = Bone volume, mm^3^; BV/TV = Bone volume fraction, %; Conn. D = Connective density, 1/mm; TMD = Tissue mineral density, mg HA/cm^3^; SMI = Structural model index (0 = parallel plates, 3 = cylindrical rods); Tb. N = Trabecular number, 1/mm; Tb. Th = Trabecular thickness, μm; Tb Sp = Trabecular separation, mm; TV = Total volume, mm^3^

**Table 2:** Cortical bone variables of stroke and control rats at ultra- distal and mid-shaft of femur.

	Stroke			Control			p-value#		
	Left / Paretic	Right / Non-paretic	STS % Diff^	Left	Right	STS % Diff^	Left	Right	STS
	n = 11	n = 10	n = 10	n = 8	n = 8	n = 8			
Ultra-distal Femur									
TV	5.7 (5.7, 6.0)	5.6 (5.6, 5.8)	-2.2 (-3.5, 2.2)	5.6 (5.5, 5.7)	6.0 (5.6, 6.1)	6.4 (1.2, 7.6)	0.05	0.25	0.03
BV	5.1 (5.0, 5.2)	4.9 (4.8, 5.0)	-2.0 (-8.4, 2.9)	5.0 (4.8, 5.1)	5.2 (5.0, 5.3)	5.6 (3.2, 6.9)	0.25	0.06	0.02
vBMD	853.2 (837.4, 869.0)	819.5 (800.8, 830.6)	-5.1 (-7.1, 3.1)	869.9 (836.4, 883.2)	863.0 (848.3, 872.5)	-0.8 (-1.0, 0.5)	0.42	0.02	0.16
TMD	967.6 (959.7, 975.2)	951.8 (943.3, 958.1)	-1.8 (-2.8, -0.4)	973.0 (960.4, 986.3)	973.4 (969.2, 981.7)	0.1 (-0.6, 1.1)	0.89	0.33	0.53
Mid-Shaft									
TV	13.8 (13.4, 14.8)	13.8 (13.4, 14.2)	1.4 (-0.2, 3.0)	14.0 (13.6, 14.2)	14.0 (13.3, 14.0)	2.3 (-0.5, 3.4)	0.05	0.25	0.03
BV	11.3 (10.8, 11.9)	11.5 (11.0, 11.6)	0.7 (0.1, 1.6)	11.6 (11.3, 11.7)	11.6 (11.1, 11.7)	1.5 (-0.1, 1.9)	0.79	0.66	0.66
vBMD	1045.1 (1035.2, 1049.2)	1053.1 (1050.2, 1059.5)	-1.1 (-4.8, 2.3)	1043.0 (1038.1, 1058.4)	1058.6 (1056.2, 1060.6)	-5.8 (-7.5, -0.1)	0.59	0.29	0.33
TMD	1125.4 (1120.8, 1131.3)	1128.8 (1123.6, 1132.6)	1.3 (-1.7, 2.6)	1126.3 (1120.8, 1132.0)	1131.3 (1130.7, 1134.8)	0.3 (-1.9, 1.4)	0.89	0.33	0.53
Ct. Ar	6.63 (6.47, 6.95)	6.64 (6.55, 6.73)§	-0.78 (-3.31, 0.07) §	6.81 (6.49, 6.83)¥	6.67 (6.37, 6.75)	-1.55 (-4.10, -0.72) ¥	0.82	0.92	0.43
Ct. Th	0.20 (0.18, 0.23)	0.23 (0.21, 0.24)§	3.03 (-2.89, 14.43) §	0.21 (0.19, 0.22) ¥	0.23 (0.22, 0.25)	12.5 (-4.12, 25.79) ¥	0.82	0.67	0.88

*Note*. Median values (IQR) presented.

^ Side-to-side % difference; calculated [(right leg–left leg)/ left leg] x 100

^#^Between-group comparison: Wilcoxon rank-sum test

^§^ n = 9,

^¥^ n = 7: data missing due to software error

BV = Bone volume, mm3; BV/TV = Bone volume fraction, %; Conn. D = Connective density, 1/mm;), Ct. Th = cortical thickness, mm,; Ct. Ar = cortical area, mm^2^) TMD = Tissue mineral density, mg HA/cm^3^; SMI = Structural model index (0 = parallel plates, 3 = cylindrical rods); Tb. N = Trabecular number, 1/mm; Tb. Th = Trabecular thickness, μm; Tb Sp = Trabecular separation, mm; TV = Total volume, mm^3^; vBMD = volumetric bone mineral density, mg HA/cm^3^

### Bone turnover markers

In control animals bone markers CTX and P1NP did not differ from baseline at either day 7 or 27, Table 3. After stroke, there was a trend towards a reduction in P1NP at day 7, reaching significance by day 27 (*p* = 0.046); no changes in CTX were observed.

**Table 3:** Bone turnover markers in rats: Percentage change from baseline.

		Stroke	Control	p-value^
		Median (IQR)	Median (IQR)	
CTX, %				
	Day 7	-7.1 (-19.1, 24.7) n = 8	1.3 (-27.1, 24.0) n = 8	0.46
	Day 27	4.9 (-5.7, 30.5) n = 9	0.7 (-34.2, 27.4) n = 7	0.79
P1NP, %				
	Day 7	-44.1 (-58.8,-26.1) n = 6	-23.8 (-35.7,-14.5) n = 6	0.42
	Day 27	-24.5 (-34.1,-10.5) n = 6^^	-0.8 (-3.4, 43.1) n = 5	0.41

CTX = Degradation products of C-terminal telopeptides of type 1 collagen, bone resorption marker. P1NP = N-terminal propeptide of type 1 procollagen, bone formation marker

^Wilcoxon rank-sum comparison between stroke and control animals

^^One-sample median test compared to baseline value, *p* = 0.046.

### Associations between bone and stroke impairments

There were no significant associations between bone density and structure of the left (paretic) femur with stroke impairments, activity or acute body weight change except between foot-fault score at day 1–2 and trabecular thickness of the left distal femur (r = 0.74, *p* = 0.01). Furthermore, no significant associations were observed between change in bone markers and stroke behavior or activity at either acute or late time points. There was an association between change from baseline in bone resorption (CTX) and sensorimotor impairment (foot-faults) of the left hindlimb at day 27 (r = 0.6, *p* = 0.014).

## Discussion

In this study we investigated the utility of a proven animal stroke model—MCAo in rats—as a model of bone loss in human stroke survivors. Our observations of group differences in bone volume and density at the distal femur and a reduction of bone formation marker P1NP in animals with stroke–despite similar physical activity patterns between groups—suggest that stroke may suppress bone formation. Based on human stroke studies [10,29], evidence from hind-limb immobilization and unloading in neurologically intact rats [5–7], and the neural control of bone metabolism [11,12,30], it was hypothesized that MCAo would compromise the microstructure of femurs on the left (paretic) leg. There was no effect of stroke observed on the primary outcome (trabecular bone volume fraction of left femurs), but there were a number of changes in secondary outcomes that may prove to be important findings and should be considered for inclusion in future studies. They may also be better candidates for choice as primary outcomes. We suggest that the ultra-distal femur site be included in future studies, given that we observed group differences at this site but not at the mid-shaft.

Bone resorption (CTX) did not change in either group, but bone formation (PINP) was reduced after stroke by day 27. Given that P1NP was reduced despite no group differences observed in activity or foot-faults, this may indicate that factors other than physical activity may have contributed to changed bone formation after stroke. Brain infarct may reduce bone formation, in keeping with studies by Vignaux et al. (2013)[30] who observed that despite no change in physical activity, rats with bilateral vestibular lesions experienced suppression of bone formation but no change in bone resorption. The authors observed an increase in the sympathetic nervous system (SNS) outflow, suggesting that bone remodeling is in part regulated by the vestibulo-sympathetic system [30]. This supported previous findings in which overstimulation of peripheral sympathetic neurons released neuropeptide Y (NPY), which in turn inhibited osteoblast function to reduce bone formation through activation of the hypothalamic Y2 receptor [31].

Our observation that stroke animals compared to controls had lower cortical vBMD and TMD, and trabecular TMD of right (non-paretic) legs, but not left (paretic) legs, was opposite to our expectations and warrants further investigation. Our results reflect previous observations of bilateral asymmetry of rat femurs [32], however Fox et al observed that rats’ left femurs were heavier than their right. Differences in the direction of asymmetry between the two animal cohorts may be explained by animals’ genetics and environmental factors [32]. Our animals’ right sided paw preference for grooming and other activities [33] and post-stroke postural control may explain some of our observations… In rats with unilateral dopamine depletion (hemi-Parksinson analogue rats) [34], reduced postural control of contralateral limbs was observed to limit rats’ reaching abilities with their “normal” limbs. It is possible that after stroke animals may have preserved the bone mass of their left (stroke affected) limbs via increased weight bearing on their left side, due to loss of postural control. The detailed examination of rats’ post-stroke movement symmetries and limb loading is warranted but was beyond the scope of this study. Furthermore, the lack of prospective data limits our understanding of limb bone mass and bone formation after stroke.

Moreover, the bone imaging method used may have been insensitive to detect subtle changes that occur at the cortical-trabecular bone interface. To better understand structural changes that occur after stroke newer technology such as StrAx software, would delineate cortical and trabecular bone regions from the cortical–trabecular interface to provide greater sensitivity of results[35]. Furthermore, analysis of a larger region of interest may provide further detail about changes that occur along the length of the bone.

To confirm a lack of association between physical activity and skeletal parameters, tools used to measure activity and impairments in animals must be valid and sensitive enough to detect subtle impairments after MCAo. Although our battery of tests is commonly used in neurological rodent models, there were no published data regarding reliability and validity of these assessments. Intra-tester reliability assessment of the foot-fault test found a substantial and significant concordance, although the strength of the relationship reduced as the number of foot-faults increased. Furthermore, there was a flooring effect observed in the foot-fault test: some of the most impaired animals did not move when placed on the ladder at day 1. Use of a test that allows scoring of even the most impaired animals is required; new guidelines on stroke impairment testing in rodents are being developed [36].

The method of physical activity recording is also important, given inconsistency in the literature about the effect of stroke on spontaneous activity in animals. Similar to the current study, activity did not change after hemorrhagic stroke in the right caudoputamen of rats [16], but hyperactivity was observed with MCAo [17,18]. On aggregate, results indicate that after stroke, activity may not change, or may increase, which is in contrast to humans with stroke who mostly remain sedentary after stroke [15]. The skeletal effect of brain injury, separate to physical activity, could be further tested in a model of total hindlimb unloading by comparing outcomes between tail suspended animals with and without stroke, and ambulant animals with and without stroke.

Another important consideration is the timing of assessments: given that resorption precedes formation in the bone remodeling cycle, it is possible that unobserved changes may have occurred in CTX between baseline and the first post-stroke measurement at 7 days after stroke. It has been recommended that the minimum interval for measuring markers of bone modelling is 2 days in rats [37], so the first post-stroke measure should be at day 2. Furthermore, given that the period of one bone remodeling cycle in rats is approximately 15–25 days [38], a study length longer than 28 days may be required to observe the full effect of changes in bone markers on microstructure [39]. It is recognized that P1NP and CTX are useful biochemical markers of bone turnover, but their use is limited due to biological variability [40]:. Although beyond the scope of this study, the examination of osteoclast and osteoblast activity on bone surfaces and bone mineralization rate is warranted to extend our understanding of bone cellular changes in response to stroke.

A limitation to this study is that although the animals at age 15 weeks had reached a period of slowed growth [41], they may have been too young to resemble bone loss patterns observed in human stroke survivors. By 26 weeks, longitudinal bone growth virtually ceases in rats despite the continued presence of a growth plate [42], therefore use of rats age 26 weeks is preferential. Furthermore, as no previous data existed to inform sample size estimates, this study was likely underpowered to detect differences. Adequately powered animal studies are vital to drive the translation of animal studies into human clinical trials [36]. Results suggest that allowing for 25% animal deaths after stroke, a sample size of 88 animals would provide 0.8 power to detect a difference in cortical bone volume at the distal femur of left legs (two tailed test, α = 0.05), at a ratio of 5:4 stroke: controls.

In summary, this study was the first investigation of the skeletal effect of ischemic stroke in rats, using the well-established model of MCAo. This model reflects the most common type of stroke in humans, and successfully aligned a number of key variables for the study of post-stroke bone loss between rats and humans: hypertension, and sensorimotor impairments on the left side after MCAo. Although the primary hypothesis of reduced BV/TV in paretic hind limbs was not observed, reduced P1NP, unrelated to impairments and activity was evident after stroke. Furthermore, the side-to-side differences in trabecular and cortical bone volumes and total volumes observed in controls were not evident in stroke animals. Results suggest that neural damage from stroke may alter bone metabolism independent of physical activity, supporting previous evidence from non-stroke studies of central control of bone metabolism. This implies that methods for preservation of bone mass in non-stroke populations are not necessarily directly transferrable to stroke survivors.

Refinements to the model are required to further examine post-stroke bone loss and separately examine the skeletal effects of altered activity and neural damage. The degree of unloading of rats’ hind limbs after MCAo, and the validity of activity monitoring, need to be determined. Based on the current findings a larger cohort of animals (n = 88) aged 26 weeks old need to be examined for longer than 28 days, and bone turnover markers should be tested at post-surgery day 2. These refinements to the model will determine its utility to model human post-stroke bone loss in the quest to reduce stroke survivors’ greatly heightened fracture risk.

## Supporting information

S1 File Raw data. This file contains data from animals’ bone, behavior and activity measurements.
(XLSX)

## References

[1] Kanis J, Oden A, Johnell O (2001) Acute and long-term increase in fracture risk after hospitalization for stroke. Stroke 32: 702–706. 11239190 10.1161/01.str.32.3.702

[2] Fisher A, Srikusalanukul W, Davis M, Smith P (2013) Poststroke hip fracture: prevalence, clinical characteristics, mineral-bone metabolism, outcomes, and gaps in prevention. Stroke Res Treat 2013: 641943 10.1155/2013/641943 24187647 PMC3800649

[3] Borschmann K (2012) Exercise protects bone after stroke, or does it? A narrative review of the evidence. Stroke Res Treat 2012: 103697 10.1155/2012/103697 22007349 PMC3189587

[4] Borschmann K, Pang M, Bernhardt J, Iuliano-Burns S (2012) Stepping towards prevention of bone loss after stroke: a systematic review of the skeletal effects of physical activity after stroke. Int J Stroke 7: 330–335. 10.1111/j.1747-4949.2011.00645.x 21967614

[5] Jamsa T, Koivukangas A, Ryhanen J, Jalovaara P, Tuukkanen J (1999) Femoral neck is a sensitive indicator of bone loss in immobilized hind limb of mouse. J Bone Miner Res 14: 1708–1713. 10.1359/jbmr.1999.14.10.1708 10491218

[6] Aguirre J, Plotkin L, Stewart S, Weinstein R, Parfitt A, et al (2006) Osteocyte apoptosis is induced by weightlessness in mice and precedes osteoclast recruitment and bone loss. Journal of Bone and Mineral Research 21: 605–615. 10.1359/jbmr.060107 16598381

[7] Laib A, Barou O, Vico L, Lafage-Proust M, Alexandre C, et al (2000) 3D micro-computed tomography of trabecular and cortical bone architecture with application to a rat model of immobilisation osteoporosis. Medical & Biological Engineering & Computing 38: 326–332. 10912350 10.1007/BF02347054

[8] Jorgensen L, Jacobsen B, Wilsgaard T, Magnus J (2000) Walking after stroke: Does it matter? Changes in bone mineral density within the first 12 months after stroke. A longitudinal study. Osteoporosis International 11: 381–387. 10.1007/s001980070103 10912838

[9] Jorgensen L, Jacobsen BK (2001) Functional status of the paretic arm affects the loss of bone mineral in the proximal humerus after stroke: a 1-year prospective study. Calcif Tissue Int 68: 11–15. 12037618 10.1007/BF02684997

[10] Pang M, Ashe M, Eng J (2007) Muscle weakness, spasticity and disuse contribute to demineralization and geometric changes in the radius following chronic stroke. Osteoporosis International 18: 1243–1252. 10.1007/s00198-007-0372-6 17401512 PMC3114013

[11] Wong I, Zengin A, Herzog H, Baldock P (2008) Central regulation of bone mass. Semin Cell Dev Biol 19: 452–458. 10.1016/j.semcdb.2008.08.001 18761098

[12] Vignaux G, Ndong J, Perrien D, Elefteriou F (2014) Inner ear vestibular signals regulate bone remodeling via the sympathetic nervous system. J Bone Miner Res 30: 1103–1111. 10.1002/jbmr.2426 PMC4772960 25491117

[13] Lee J, Kim J, Kim H, Choi E, Lim S, et al (2005) Changes in bone metabolism in a rat model of traumatic brain injury. Brain Injury 19: 1207–1211. 10.1080/02699050500309338 16286336

[14] Freret T, Chazalviel L, Roussel S, Bernaudin M, Schumann-Bard P, et al (2006) Long-term functional outcome following transient middle cerebral artery occlusion in the rat: correlation between brain damage and behavioral impairment. Behav Neurosci 120: 1285–1298. 10.1037/0735-7044.120.6.1285 17201474

[15] English C, Manns P, Tucak C, Bernhardt J (2014) Physical activity and sedentary behaviors in people with stroke living in the community: a systematic review. Phys Ther 94: 185–196. 10.2522/ptj.20130175 24029302

[16] Snow L, Low W, Thompson L (2012) Skeletal muscle plasticity after hemorrhagic stroke in rats: influence of spontaneous physical activity. Am J Phys Med Rehabil 91: 965–976. 10.1097/PHM.0b013e31825f18e1 22760110

[17] Quinn L, Grundy R, Campbell C, Collier S, Lawman A, et al (2005) A novel behavioural registration system LABORAS and the social interaction paradigm detect long-term functional deficits following middle cerebral artery occlusion in the rat. Brain Res 1031: 118–124. 10.1016/j.brainres.2004.10.036 15621019

[18] Borlongan C, Cahill D, Sanberg P (1995) Locomotor and passive avoidance deficits following occlusion of the middle cerebral artery. Physiology & behavior 58: 909–917. 8577887 10.1016/0031-9384(95)00103-p

[19] Kilkenny C, Browne W, Cuthill IC, Emerson M, Altman DG (2010) Animal research: reporting in vivo experiments: the ARRIVE guidelines. Br J Pharmacol 160: 1577–1579. 10.1111/j.1476-5381.2010.00872.x 20649561 PMC2936830

[20] Rewell S, Fernandez J, Cox S, Spratt N, Hogan L, et al (2010) Inducing stroke in aged, hypertensive, diabetic rats. J Cereb Blood Flow Metab 30: 729–733. 10.1038/jcbfm.2009.273 20068574 PMC2949155

[21] Chiang C, Chiu M, Moore A, Anderson P, Ghasem-Zadeh A, et al (2009) Mineralization and bone resorption are regulated by the androgen receptor in male mice. J Bone Miner Res 24: 621–631. 10.1359/jbmr.081217 19049333

[22] Bouxsein M, Boyd S, Christiansen B, Guldberg R, Jepsen K, et al (2010) Guidelines for assessment of bone microstructure in rodents using micro-computed tomography. J Bone Miner Res 25: 1468–1486. 10.1002/jbmr.141 20533309

[23] Petullo D, Masonic K, Lincoln C, Wibberley L, Teliska M, et al (1999) Model development and behavioral assessment of focal cerebral ischemia in rats. Life Sciences 64: 1099–1108. 10210272 10.1016/s0024-3205(99)00038-7

[24] Metz G, Whishaw I (2009) The ladder rung walking task: a scoring system and its practical application. J Vis Exp. 10.3791/1204 PMC2796662 19525918

[25] Batchelor P, Kerr N, Gatt A, Aleksoska E, Cox S, et al (2010) Hypothermia prior to decompression: buying time for treatment of acute spinal cord injury. J Neurotrauma 27: 1357–1368. 10.1089/neu.2010.1360 20504158

[26] Bolton D, Tse A, Ballermann M, Misiaszek J, Fouad K (2006) Task specific adaptations in rat locomotion: runway versus horizontal ladder. Behavioural Brain Research 168: 272–279. 10.1016/j.bbr.2005.11.017 16406145

[27] Stryjek R, Modlinska K, Turlejski K, Pisula W (2013) Circadian rhythm of outside-nest activity in wild (WWCPS), albino and pigmented laboratory rats. PLoS One 8: e66055 10.1371/journal.pone.0066055 23762462 PMC3676357

[28] Landis J, Koch G (1977) The measurement of observer agreement for categorical data. Biometrics 33: 159–174. 843571

[29] Jorgensen L, Jacobsen B (2001) Changes in muscle mass, fat mass, and bone mineral content in the legs after stroke: A 1 year prospective study. Bone 28: 655–659. 11425655 10.1016/s8756-3282(01)00434-3

[30] Vignaux G, Besnard S, Ndong J, Philoxene B, Denise P, et al (2013) Bone Remodeling Is Regulated by Inner Ear Vestibular Signals. Journal of Bone and Mineral Research 28: 2136–2144. 10.1002/jbmr.1940 23553797

[31] Driessler F, Baldock P (2010) Hypothalamic regulation of bone. J Mol Endocrinol 45: 175–181. 10.1677/JME-10-0015 20660619

[32] Fox KM, Kimura S, Plato CC, Kitagawa T (1995) Bilateral asymmetry in bone weight at various skeletal sites of the rat. Anat Rec 241: 284–287. 10.1002/ar.1092410215 7710144

[33] Windle V, Corbett D (2005) Fluoxetine and recovery of motor function after focal ischemia in rats. Brain Res 1044: 25–32. 10.1016/j.brainres.2005.02.060 15862786

[34] Miklyaeva E, Woodward N, Nikiforov E, Tompkins G, Klassen F, et al (1997) The ground reaction forces of postural adjustments during skilled reaching in unilateral dopamine-depleted hemiparkinson rats. Behav Brain Res 88: 143–152. 9404623 10.1016/s0166-4328(97)00043-0

[35] Zebaze R, Ghasem-Zadeh A, Mbala A, Seeman E (2013) A new method of segmentation of compact-appearing, transitional and trabecular compartments and quantification of cortical porosity from high resolution peripheral quantitative computed tomographic images. Bone 54: 8–20. 10.1016/j.bone.2013.01.007 23334082

[36] Bernhardt J, Borschmann K, Boyd L, Carmichael ST, Corbett D, et al (2016) Moving rehabilitation research forward: Developing consensus statements for rehabilitation and recovery research. International Journal of Stroke: 1747493016643851. 10.1177/1747493016643851 27073187

[37] Erben R (2003) Handbook of Histology Methods for Bone and Cartilage; An Y, Martin K, editors.

[38] Erben RG, Eberle J, Stahr K, Goldberg M (2000) Androgen deficiency induces high turnover osteopenia in aged male rats: a sequential histomorphometric study. J Bone Miner Res 15: 1085–1098. 10.1359/jbmr.2000.15.6.1085 10841177

[39] Dion N, Fortin A, Ste-Marie L (2011) Methods in Bone Histomorphometry for Animal Models In: Duque G, Watanabe K, editors. Osteoporosis Research: Animal Models. London: Springer-Verlag pp. 38.

[40] Vasikaran S, Eastell R, Bruyere O, Foldes AJ, Garnero P, et al (2011) Markers of bone turnover for the prediction of fracture risk and monitoring of osteoporosis treatment: a need for international reference standards. Osteoporos Int 22: 391–420. 10.1007/s00198-010-1501-1 21184054

[41] Hughes PC, Tanner JM (1970) The assessment of skeletal maturity in the growing rat. J Anat 106: 371–402. 4315144 PMC1233709

[42] Roach H, Mehta G, Oreffo R, Clarke N, Cooper C (2003) Temporal analysis of rat growth plates: cessation of growth with age despite presence of a physis. J Histochem Cytochem 51: 373–383. 10.1177/002215540305100312 12588965

